# Neuroinflammation and progressive myoclonus epilepsies: from basic science to therapeutic opportunities

**DOI:** 10.1017/erm.2020.5

**Published:** 2020-09-17

**Authors:** Pascual Sanz, José M. Serratosa

**Affiliations:** 1 Instituto de Biomedicina de Valencia (CSIC) and Centro de Investigación Biomédica en Red de Enfermedades Raras (CIBERER), Jaime Roig 11, 46010 Valencia, Spain; 2 IIS Fundación Jimenez Diaz and Centro de Investigación Biomédica en Red de Enfermedades Raras (CIBERER), Madrid, Spain

**Keywords:** Lafora disease, lysosome, mitochondrial dysfunction, neuroinflammation, neuronal ceroid lipofuscinosis, PMEs, progressive myoclonus epilepsy, Unverricht-Lundborg disease

## Abstract

Progressive myoclonus epilepsies (PMEs) are a group of genetic neurological disorders characterised by the occurrence of epileptic seizures, myoclonus and progressive neurological deterioration including cerebellar involvement and dementia. The primary cause of PMEs is variable and alterations in the corresponding mutated genes determine the progression and severity of the disease. In most cases, they lead to the death of the patient after a period of prolonged disability. PMEs also share poor information on the pathophysiological bases and the lack of a specific treatment. Recent reports suggest that neuroinflammation is a common trait under all these conditions. Here, we review similarities and differences in neuroinflammatory response in several PMEs and discuss the window of opportunity of using anti-inflammatory drugs in the treatment of several of these conditions.

## Introduction

Progressive myoclonus epilepsies (PMEs) are a group of neurological disorders characterised by the occurrence of epileptic seizures, myoclonus and progressive neurological deterioration, including cerebellar involvement and dementia (Refs 1–5). PMEs include more than a dozen different diseases that are classified as rare diseases because each of them affects less than 1:2000 individuals.

The most common PME's are: (1) Unverricht-Lundborg disease [ULD; Epilepsy Progressive Myoclonus 1 (EPM1) (OMIM #254800)], because of mutations in the *CSTB* gene encoding cystatin B, a lysosomal cysteine protease inhibitor; (2) Lafora disease (LD; EPM2) (OMIM #254780), because of mutations in either *EPM2A* gene, encoding the glucan phosphatase laforin, or *EPM2B* gene, encoding the E3-ubiquitin ligase malin; (3) the neuronal ceroid lipofuscinoses (NCLs), a collection of disorders because of mutations in more than 10 different *CLN* genes; (4) Sialidosis, a lysosomal storage disease because of mutations in the *NEU1* gene encoding the lysosomal enzyme alpha-*N*-acetylneuraminidase (sialidase) (OMIM #256550) and (5) Myoclonic epilepsy with ragged fibres (MERRF), because of mutations in the mitochondrial gene *MT-TK* encoding tRNA^Lys^ (OMIM #54500) (Ref. 5) (Table 1).

**Table 1.** Description of the most common PMEs covered in this work

PME (acronym)	Affected genes	Protein function
Unverricht-Lundborg disease (ULD)	*CSTB*	Cystatin B, a cathepsin B lysosomal protease inhibitor
Lafora disease (LD)	*EPM2A*, *EPM2B*	Laforin, a glucan phosphatase; Malin, an E3-ubiquitin ligase
Neuronal ceroid lipofuscinosis (NCLs)	Several *CLN* genes	Cln1/Ppt1, lysosomal palmitoyl protein thioesterase; Cln2/Tpp1, lysosomal tripeptidyl peptidase 1; Cln3, a lysosomal membrane protein; Others
Sialidosis (ST-1)	*NEU1*	Neu1, lysosomal alpha-N-acetylneuraminidase
Myoclonic epilepsy with ragged fibres (MERRF)	*MT-TK*	Mitochondrial lysine transfer tRNA

Although the primary cause of PMEs is different in each case, here we review the similarities and differences in the neuroinflammatory response in several PMEs and discuss the window of opportunity of anti-inflammatory drugs in the treatment of several of these conditions.

## Unverricht-Lundborg disease (ULD, EPM1, OMIM #254800)

The onset of ULD is around late childhood and early adolescence. It is characterised by action myoclonus and generalised tonic-clonic seizures that may occur without prior myoclonic jerks. Generalised tonic-clonic seizures occur typically on awakening or during sleep. As the disease progresses, the myoclonus increases in intensity and frequency, and in more severely affected patients, it causes major disability forcing patients to be wheelchair-bound or even bedridden. ULD also progresses to include associated neurological symptoms such as ataxia, impaired gait and cognitive impairment. On contrary to other PMEs, early death is not common in ULD and the outcome of adult patients ranges from minimal impairment with an independent active life to severe disability (Refs 6, 7).

ULD is an autosomal recessive disorder caused by mutations in the gene encoding cystatin B/Stefin B (*CSTB*), an 11 kDa inhibitor of lysosomal cathepsin B protease (Table 1). The most common mutation is an expansion of a minisatellite sequence repeat (CCCCGCCCCGCG) in the 5′-untranslated region of the *CSTB* gene. An expansion of 30–80 repeats is causative and leads to reduced expression of the gene (Ref. 7). In addition, frameshift mutations and deletions are also found among ULD patients, although they are less common (Ref. 6). ULD is characterised by a loss of cerebellar granular neurons, although a clear activation of microglia precedes neuronal loss (Refs 8, 9).

A mouse model of ULD that lacks the *CSTB* gene (*Cstb-*/*-*) shows myoclonic seizures, ataxia and progressive neuronal loss together with cerebellar and cortical atrophy that aggravates with age. Although 2-week old mice are asymptomatic, they already present signs of microglial activation. Activation of microglia is considered as the trigger to astrocyte reactivity, neuroinflammation and progressive neuronal loss (Ref. 8). Interestingly, double *Cstb-*/*- cathepsin B-*/*-* knockout mice still present seizures and ataxia, suggesting that cystatin B may have other functions in cell physiology in addition to being an inhibitor of lysosomal cathepsin B protease (Ref. 8).

In order to identify the transcriptomic signature related to ULD, RNA was extracted from the brain of *Cstb-*/*-* mice of 1 month age and analysed by microarray techniques (Ref. 10). The authors indicated that there was an upregulation of genes related to immune response and defense, such as those encoding complement proteins (*C1qa*, *C1qb*, *C1qc*, *C4b* and *C3ar1*), major immunohistocompatibility complex class I (MHC-I), b2-microglobulin (*B2m*), glial fibrillary acidic protein (*Gfap*), chemokines (*Cxcl13* and *Ccl6*), immunoglobulin receptors (*Fcgr3* and *Fcgr1g*), cluster differentiation antigens (*Cd14*, *Cd44*, *Cd48* and *Cd52*), and components of lytic vacuoles [*HexB* (hexosaminidase B), *Cstd* (cathepsin D), *Csth* (cathepsin H) and Cd68 antigen], among others (Ref. 10). In light of these results, the authors proposed that early-onset neuroinflammation was key in the pathogenesis of ULD. They also proposed that glial-derived proinflammatory chemokines and cytokines contributed to recurrent excitation and epilepsy (Ref. 10). The increased production of proinflammatory cytokines and chemokines was because of the activation of the non-canonical pathway of the inflammasome as *Cstb-*/*-* mice presented increased levels of the mediators of this route, namely caspase11 and gasdermin D (Ref. 11). All these changes lead to an increased sensitivity of microglia from *Cstb*-/- mice to LPS, which stimulated a higher production of different cytokines and chemokines (Ref. 12). In fact, an RNAseq analysis of primary cultures of microglia from *Cstb-*/*-* mice of 5 days of age demonstrated an upregulation of genes related to immune and defense response, interferon signalling and antigen presentation (Ref. 13). The authors also indicated that the gene ontology (GO) molecular functions of the upregulated genes were nucleotide-binding (Oas1a, Oas1b and Oas2; Oasl1, Oasl2; Schafen proteins Slfn2 and Slfn5), GTPase activity (Mx1, Gbp1/2, Irgm1/2 and Gvin1) and chemotaxis (Ccl2, Ccl5, Cxcl10 and Cxcl13) (Ref. 13).

The neuroinflammation present in the brain of *Cstb-*/*-* mice is accompanied by peripheral inflammation demonstrated by higher levels of chemokines (Cxcl1, Cxcl10 and Cxcl13) and cytokines (IL-1a and IL18) in serum, and the authors proposed the levels of Cxcl13 as a biomarker of the disease (Ref. 9).

In the absence of *Cstb*, there is also a clear mitochondrial dysfunction, with decreased membrane potential and increased reactive oxygen species (ROS) production, which could stimulate an inflammatory response. The authors suggest that this mitochondrial dysfunction could be the primary cause of the pathophysiology of ULD (Refs 14, 15).

## Lafora disease (LD, EPM2, OMIM #254780)

As in the case of ULD, the onset of LD is also around late childhood and early adolescence. It is characterised by the appearance of generalised tonic-clonic seizures, myoclonus, absences and visual hallucinations. The disease progresses rapidly with a worsening of seizures and dementia, leading to the death of the patient after a decade from the onset of the first symptoms. The hallmark of LD is the accumulation of insoluble poorly branched glycogen deposits in the brain and peripheral tissues, known as Lafora bodies (LBs) (Refs 16, 17).

LD is also an autosomal recessive disorder caused by mutations in the *EPM2A* gene, encoding the 37 kDa glucan phosphatase laforin, or the *EPM2B* gene, encoding the 42 kDa E3-ubiquitin ligase malin. Both proteins form a functional complex and perhaps this is the reason why mutations that affect the activity of any of the two components, or mutations that affect the interaction between them but preserve their intrinsic activities, are pathogenic and lead to similar pathophysiological presentations (Refs 17, 18). It has been described that the laforin/malin complex plays a negative role in the regulation of glycogen biosynthesis: the laforin/malin complex ubiquitinates several glycogenic enzymes such as glycogen synthase, glycogen debranching enzyme, protein targeting to glycogen, etc., and maintains glycogen synthesis at low levels. In the absence of a functional laforin/malin complex glycogen synthesis is enhanced leading to the production of LBs (Refs 19, 20).

In order to study the pathophysiology of LD, several animal models have been used: *Epm2a-*/*-* mice lack exon 4 from the *Epm2a* gene (Ref. 21) and *Epm2b-/-* mice lack the single exon present in the *Epm2b* gene (Refs 22–24). Both mouse models present similar pathophysiological phenotypes, that is, they show similar behavioural impairments (Ref. 25), are more sensitive to the effects of the pro-epileptic drug pentylenetetrazole (Ref. 26), and accumulate LBs in the brain and other peripheral tissues (Refs 22–24). Using these animal models, it was proposed that the accumulation of LBs was the primary cause of the disease as in LD mice unable to synthesise glycogen because of a deletion of genes involved in glycogen synthesis (e.g. *GYS1* or *PPP1R3C*), no LBs were formed and animals presented no signs of disease (Refs 27–30).

It seems that the accumulation of LBs is deleterious to the cell and affects different physiological pathways, for example, LD mice present altered autophagy and mitophagy (Refs 24, 31–33) and signs of oxidative stress (Ref. 34). In these LD mouse models, the disease progresses with age, being the pathological phenotypes more severe as the animals get older (Refs 21, 25, 35).

We have recently reported additional traits in LD, namely neuroinflammation. We and others initially mentioned the presence of reactive astrocytes and microglia in the brain of LD animals (Refs 23, 27, 35, 36), and we have recently described that, in the brain of LD animals, there is an upregulation of the expression of a full set of proinflammatory genes, ranging from immune system response to inflammatory response and phagocytosis (Ref. 37). Interestingly, among the GO terms related to the upregulated genes we found: (i) cytokines and their receptors (e.g. IL-1b, IL-1a and IL1rl1); (ii) chemokines and their receptors (e.g. Cxcl10, Ccl2 and Ccl5); (iii) complement proteins (e.g. C1qa, C1qb, C1qc, C3 and C4b); (iv) Toll-like receptors (e.g. Tlr1, Tlr2 and Tlr7), (v) inflammasome related proteins (e.g. Casp4, Naip2, Naip5 and Naip6); (vi) components of the major histocompatibility complex classes I and II; (vii) immunoglobulin receptors (e.g. Fcgr1–4 and Clec7a), (viii) cluster differentiation antigens (e.g. Cd14, Cd44, Cd48 and Cd52); (ix) phagocytosis related components (e.g. Trem2, Tyrobp and Cd68) and (x) cytoskeleton proteins (e.g. Gfap and vimentin); among others. Surprisingly, the fold change increase in the expression of these genes was similar in *Epm2a-*/*-* and *Epm2b-*/*-* mice, suggesting that both were responsible for the similar pathophysiological phenotype presented in these two models of LD. We also observed that these genes were expressed mainly by reactive astrocytes and microglia, suggesting a major role of these cells in the progress of neuroinflammation. In addition, we observed that the upregulation of these genes correlated with age and with the worsening of the disease. These results clearly indicated that neuroinflammation is an important trait to be considered in order to fully understand the pathophysiology of LD (Ref. 37).

Both mouse models of LD also presented mitochondrial dysfunction with a decrease in membrane potential, an increase in ROS production and a decrease in the activity of typical anti-oxidant enzymes (Ref. 34). In addition, because of impairment in autophagy, defective mitochondria were not removed by mitophagy, worsening mitochondrial dysfunction (Ref. 33). Therefore, as in the case of ULD, there is a correlation between the presence of oxidative stress and neuroinflammation in this form of PME.

## Neuronal ceroid lipofuscinosis (Batten disease, NCLs)

NCLs are a group of fatal lysosomal storage disorders. Each form of NCL is caused by mutations in a different gene (*CLN1*, OMIM #256730; *CLN2*, OMIM #204500; *CLN3*, OMIM #204200; *CLN4*, OMIM #204300; *CLN5*, OMIM #256731; *CLN6*, OMIM #601780; *CLN7*, OMIM #610951; *CLN8*, OMIM #600143; *CLN9*, OMIM #609055; *CLN10*, OMIM #610127; *CLN11*, OMIM #614706; *CLN12*, OMIM #256730 and *CLN13*, OMIM #615362), which determines the progression and severity of the disease, although they all lead to the death of the patient after a period of prolonged disability (Refs 38–40). NCLs are characterised by a progressive decline of cognitive and motor abilities, retinopathy evolving into blindness, cerebellar atrophy and myoclonic epilepsy, leading to decreased life expectancy (reviewed in Ref. 5). All NCLs are characterised by the accumulation inside the lysosome of autofluorescent storage material and major neuronal loss, although no direct relationship between these two characteristics has been found yet and the idea that pathology was a consequence of storage body accumulation is no longer tenable (Refs 41, 42).

However, in all forms of NCLs, reactive astrocytes and microglia are detected at early stages of the disease and, as the disease progresses, there is a direct correlation between the magnitude of glial reactivity and the neuronal loss (Refs 41, 43–45).

The most common form of NCL, juvenile NCL, is because of mutations in the *CLN3* gene (OMIM #204200). *CLN3* encodes battenin, a 47 kDa lysosomal membrane protein involved in microtubule-involved movement of endosomes and lysosomes (Ref. 46). Using a *Cln3* deficient mouse model, it was demonstrated that astrocytes and microglia are key players in the development of NCL. Reactive astrocytes in *Cln3-*/*-* mice show disrupted actin and intermediate filament cytoskeleton and an impaired ability to propagate Ca^2+^ signals. They also present deficient clearance of glutamate from the synaptic cleft, suggesting an impairment in neuron-glia communication in the NCL-affected brain (Ref. 41). Using co-cultures of astrocytes and neurons, the authors elegantly showed that the presence of *Cln3-*/*-* astrocytes was able to harm control neurons in the co-cultures. This is another example of how reactive astrocytes primed by microglia may directly harm neurons (Ref. 47). Therefore the primary cause of NCL-CLN3 disease is an alteration of the functionality of the astrocytes rather than a problem at the neuronal level. Similar results were reported recently in another model of NCL, carrying a defective *CLN1* gene (OMIM #256730) (Ref. 45).

A link between the NCLs and neuroinflammation has also been reported. The analysis of brain tissue from *Cln1-*/*-* (*Ppt1-*/*-*) mice (lacking the 34 kDa lysosomal palmitoyl protein thioesterase, Ppt1) as well as from NCL-CLN1 disease patients indicated an increase in the amount of receptors for advanced glycation end products (RAGE) and activation of the NF-κB pathway, resulting in the production of proinflammatory cytokines (IL-1b, IL-6 and TNFa) and chemokines (Ccl2), which most likely contributes to neuroinflammation in NCL-CLN1 disease (Ref. 48). Recently, a proteomic analysis of brain and cerebrospinal fluid from *Cln1-*/*-*, *Cln2-*/*-* (lacking the 61 kDa lysosomal tripeptidyl peptidase 1, Tpp1; OMIM #204500) and *Cln3-*/*-* mouse models have revealed an upregulation in the levels of (i) proteins related to lysosomal function (Arsa, CD63, Ctsa, Ctsd, Ctsz, Hexb, Fuca1 and Gns); (ii) inflammatory response (GPNMB, CD44, LYZ2, SERPINA3N, GFAP, AIF1/IBA1, APOE, Capg, Cpne1, Gbp2, Ifit3, Irgm1, itgb2 and Stat1) and (iii) complement proteins (C1qa, C1qb, C1qc, C4b, CD44 and S100A6) (Ref. 49). All these data suggest that glial-derived neuroinflammation could underlie the pathophysiology of the NCLs.

## Similarities and dissimilarities in the neuroinflammatory response in progressive myoclonus epilepsies

**Fig. 1.**
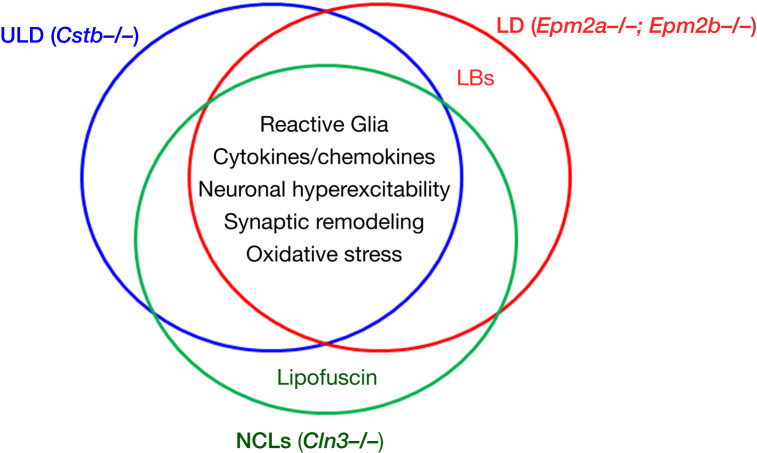
Schematic view of the similarities between ULD, LD and NLCs in terms of pathophysiological mechanisms. In the intersection, we describe the main features present in the three disorders, according to the data obtained with animal models of the corresponding diseases. The hallmark of the corresponding disease is also indicated. LBs, Lafora bodies. See text for details.

Neuroinflammation is a common trait in neurodegenerative disorders, from the most common (Alzheimer, Parkinson, etc.) to the less frequent ones (leukodystrophies, etc.) (see Ref. 50 for review). It is becoming clear that brain inflammation promotes neuronal hyper-excitability and seizures, and that dysregulation in the glia immune-inflammatory function is a common factor that predisposes or contributes to the generation of seizures. At the same time, acute seizures upregulate the production of pro-inflammatory cytokines in microglia and astrocytes, triggering a downstream cascade of inflammatory mediators. Therefore, epileptic seizures and inflammatory mediators form a positive feedback loop, reinforcing each other (Ref. 50). As it has been described above, the recurrent theme in three forms of PMEs (ULD, LD and NCLs) is the presence in the brain of reactive glia (astrocytes and microglia), which appears at early stages of the disease, even before neuronal degeneration is observed. At present, it is not clear what triggers glial activation in each case. It could be the accumulation of intracellular deposits, as in LD and the NCLs, which could act as danger-associated molecular patterns to activate inflammatory pathways (Fig. 1; Table 2). Alternatively, mitochondrial dysfunction accompanied by ROS production (oxidative stress), in the case of ULD and LD, could be the trigger of the initial inflammatory response (Fig. 1). In any case, it is becoming clear that an initial insult could activate astrocytes and/or microglia and prime their general activation which would then lead to neuronal degeneration (Table 2). This is an important mechanism that enhances the importance of astrocytes and microglia in these diseases. It points to astrocytes and microglia as the primary cause of the disease as opposed to the neurocentric hypothesis of initial neuronal problems. However, at the moment, it is not possible to define an ‘order of events’ in the neuroinflammatory process because astrocytes and microglia are so interconnected that the activity of one type of cell affects the activity of the other (Refs 51, 52).

**Table 2.** Genes and proteins upregulated in the brain of ULD, LD and NCL mouse models covered in this study

	ULD (*Cstb-*/*-* mice) (Refs 6, 9, 10, 11, 13, 15)	LD (*Epm2a-*/*-*, *Epm2b-*/*-* mice) (Refs 35, 37)	NCLs (*Cln1-*/*-*, *Cln2-*/*-*, *Cln3-*/*-* mice) (Refs 41, 48, 49)
Reactive astrocytes (GFAP+)	Yes	Yes	Yes
Activated microglia (AIF1/IBA1+)	Yes	Yes	Yes
Cytokines	IL-1a, IL-1b, IL-18	IL-1a, IL1b, IL1rl1	IL1b, IL-6, TNFa
Chemokines	Ccl2, Ccl5, Ccl6, Cxcl1, Cxcl10, Cxcl13	Ccl2, Ccl5, Ccl6, Ccl8, Ccl12, Cxcl5, Cxcl10, Cxcl16	Ccl2
Complement proteins	C1qa, C1qb, C1qc, C4b, C3ar1	C1qa, C1qb, C1qc, C1s1, C3, C4b, C3ar1	C1qa, C1qb, C1qc, C4b
Immunoglobulin receptors	Fcgr1g, Fcgr3	Clec7a, Fcgr1-4, Itgax, Lgals3	Nd
Cluster differentiation antigens	Cd14, Cd44, Cd48, Cd52	Cd14, Cd44, Cd48, Cd52, GPNMB, Lyz2, Serpina3A, among others	Cd44, GPNMB, Lyz2, Serpina3N
Major histocompatibility complex (MHC-I)	Yes	Yes	Nd
Lysosomal components	CtsD, CtsH, Cd68, HexB	Cd68, CtsZ, HexB	Arsa, Cd63, CtsA, CtsD, CtsZ, Fuca1, Gns, HexB
Phagocytosis components	Nd	Trem2, Tyrobp, Pilra	Nd
Inflammasome components	Casp4, Gsdmd	Casp4, Naip2, Naip5, Naip6	Nd
Nucleotide binding proteins	Oas1a-b, Oas2, Oasl1-2, Slfn2 and Slfn5	Oas1a-b, Oas2, Oasl1-2, Slfn2 and Slfn5, Gna15	Nd
GTPases	Mx1, Gbp1/2, Irgm1/2, Gvin1	Arhgap9,45, Mx1, Gbp1,2,6,8,10 Irgm1/2, Gimap3-4, Rasal3	Nd
Mitochondrial dysfunction (ROS)	Yes	Yes	Nd
Dysfunctional Ca^2+^ signalling	Yes	Nd	Yes
Neuronal hyperexcitability	Decreased GABAergic inhibition	Increased Glutamatergic transmission	Increased Glutamatergic transmission
Insoluble deposits	None	Polyglucosans (LBs)	Autofluorescent deposits

Characteristic histological features are also indicated. Nd, not determined.

When compared, the proinflammatory mediators detected in ULD, LD and the NCLs are surprisingly similar (Table 2). Similar cytokines and chemokines are overproduced (e.g. IL-1a, IL-1b and Ccl2), although some of them seem to be specific of each disease, for example, Cxcl13 for ULD (Ref. 9) and Cxcl10 for LD (Ref. 37) (Fig. 1). It is also striking that similar components of the complement system are overexpressed in the three forms of PMEs (C1qa, C1qb, C1qc and C4b). Because these proteins have been involved in the recognition of synaptic terminals that need to be removed by microglia, it could be that synaptic remodelling is altered in these PMEs, as has already been demonstrated in ULD (Ref. 10) and the NCLs (Ref. 41) (Fig. 1). Of notice is also the presence of components of the inflammasome pathway in the cases of ULD and LD. Perhaps this system could be involved in triggering the inflammatory response in these PMEs. ULD and LD also present dysfunctional mitochondria with altered membrane potential and increased ROS production and dysfunctional Ca^2+^ signalling. These two defects could enhance neuroinflammation. Finally, ULD, LD and the NCLs present neuronal hyperexcitability, because of decreased GABAergic inhibition (as in ULD) or to increased glutamatergic transmission (as in LD and NCLs) because of a dysfunction in glutamate transport, which could lead to an increased amount of glutamate in the synaptic cleft and, as a consequence, in hyperexcitability. Because hyperexcitability is linked to epilepsy, perhaps the elevated levels of glutamate in the brain of LD and NCLs animal models or the inhibition of GABAergic neurons in ULD could be the underlying cause of seizures in these PMEs (Fig. 1).

Recently, excellent reviews have covered the possibility of using biomarkers to define the progression of epilepsy. For example, the levels of GFAP, S100b, TNFa, cytokines, chemokines and HMGB1 have been determined in the samples of CSF and/or blood from patients with epilepsy (Refs 50, 53). However, to our knowledge, in the cases of the PMEs covered in this review, only the levels of the chemokine Cxcl13 have been suggested as a possible biomarker for ULD (Ref. 9).

## Treatment strategies in ULD, LD and NCLs. A window of opportunity for anti-inflammatory drugs

ULD, LD and the NCLs have no specific treatment yet. Seizures and myoclonus are treated with regular anti-seizure drugs (ASDs). ASDs are initially effective although sooner they lose efficiency and patients become resistant to them.

Recently, specific treatments are being developed for these conditions. In one case of ULD because of a splicing mutation c.66G>A in exon 1, an antisense oligonucleotide (ASO) therapeutic strategy allowed the restoration of the normal splicing pattern, leading to a recovery of the disease that was dose-specific. This adds evidence to the feasibility of ASO therapies and highlights the importance of personalised treatment of ULD patients (Ref. 54). As one of the pathological determinants of ULD is the presence of oxidative stress (see above), some reports have recently indicated that ULD patients treated with high doses of *N*-acetyl-cysteine showed marked improvement in seizures, ataxia and blockade of symptoms progression (reviewed in Ref. 55), suggesting that an anti-oxidant therapy could be beneficial in these disorders.

In the case of LD, the LECI consortium (Ref. 56) is working in new strategies designed to decrease the levels of polyglucosans in the brain of LD patients. One strategy is to identify new chemical compounds that inhibit glycogen synthase, the enzyme in charge of glycogen synthesis. A second strategy is the use of ASOs to inhibit the expression of the glycogen synthase gene (*GYS1*), and the third strategy is to administrate an antibody-enzyme fusion with alpha-amylase activity that could digest polyglucosans (Ref. 57). However, the efficacies of all these strategies need to be proven through a clinical trial that is being organised at the moment. In the meantime, metformin, a drug normally used for the treatment of type 2 diabetes, has been approved by the European Medicine Agency (EMA) and the Food and Drug Administration (FDA) agencies as an orphan drug for the treatment of LD (Ref. 58) and recent results suggest a positive effect of this compound in the progression of the disease (Ref. 59). It is known that metformin is an indirect activator of the AMP-activated protein kinase (AMPK), a key metabolic sensor, but as this compound has also some AMPK-independent effects (Ref. 60), it is not clear at the moment which is the actual molecular mechanism by which metformin has beneficial effects in LD.

In the NCLs, the most promising approach is enzyme replacement therapy. Intrathecal administration of recombinant PPT1 (palmitoyl protein thioesterase 1) proenzyme to the lumbar spinal cord ameliorated the pathophysiological symptoms of *Cln1* deficient mice (Ref. 61). In addition, both EMA and FDA have approved the intracerebroventricular administration of enzyme replacement therapy [with cerliponase alpha, a human pro-enzyme of TPP1 (tripeptidyl peptidase 1)] for CLN2 (Refs 62, 63). A similar strategy has recently been described using enzyme replacement therapy with recombinant human pro-cathepsin D, which when administered intracranially in *Ctsd-*/*-* mice corrected the neuronal pathophysiology of NCL-CLN10 (OMIM #610127) (Ref. 64).

As neuroinflammation is a hallmark of many PMEs, this offers a window of opportunity for the use of anti-inflammatory drugs under these conditions. This possibility is supported by recent reports that clearly state that neuroinflammatory pathways may serve as treatment targets and biomarkers in different forms of epilepsy (Refs 50, 65). In fact, it has been demonstrated that anti-inflammatory interventions in animal models of epilepsy have both anti-epileptogenic and disease-modifying therapeutic effects (Refs 50, 65). However, it has also been stated that general anti-inflammatory drugs should not be used because of their wide central and peripheral effects (Ref. 66) and that the anti-inflammatory strategy should be based on the signalling pathways that are altered under each epileptic condition. Some of these specific compounds are already in clinical use for the treatment of autoimmune diseases, so the use of specific brain-penetrant anti-inflammatory compounds that are used in other pathologies could be repurposed for drug-resistant epilepsies (Ref. 50). Therefore, the use of specific anti-inflammatory compounds is an alternative therapeutic strategy that should be explored for the treatment of the PMEs. In this sense, several efforts have already been aimed to the use of immunomodulators to decrease neuroinflammation and neurodegeneration in NCLs (Ref. 63): oral administration of fingolimod (a regulator of sphingosine-1P receptors that acts as immunosuppressor) and teriflunomide (an immunomodulatory drug that inhibits pyrimidine de novo synthesis by blocking the enzyme dihydroorotate dehydrogenase) reduced microgliosis, neuronal loss and brain atrophy in *Cln1-*/*-* and *Cln3-*/*-* mice (Ref. 67). In the same way, intraperitoneal administration of the anti-inflammatory small molecule MW151 (Ref. 68) into *Cln1-*/*-* mice decreased the incidence of seizures (Ref. 69). However, other immunosuppressive treatments failed to have a clinical effect on *CLN3* patients (Ref. 70).

As the neuroinflammation observed in the PMEs covered in the review progresses rapidly, we propose that the timing of intervention should be as early in the disease as possible to obtain better outcomes.

In conclusion, in addition to the occurrence of focal and generalised seizures, myoclonus and progressive neurological deterioration, the PMEs present neuroinflammation as a common hallmark. This opens the possibility of using specific anti-inflammatory compounds to ameliorate the pathophysiology, natural history and quality of life of patients. As most of these drugs are already used in clinical practice for other disease conditions, obtaining the designation of these compounds as orphan drugs for their use in the PMEs by the European Medicines Agency (EMA) and/or the Food and Drug Administration (FDA), could be much easier.
